# Determination of Biological Activity of Recombinant Reteplase Using Clot Lysis Time and Activated Partial Thromboplastin Time (APTT) Lysis Methods: A Comparative Study

**Published:** 2018

**Authors:** Tahereh Babaee, Ahmad Fazeli, Sameereh Hashemi-Najafabadi, Hosein Rastegar, Ali Mohammadi, Mohammad Reza Khoshayand, Mahmoud Alebouyeh, Mohammad Reza Fazeli

**Affiliations:** a *Department of Drug and Food Control, Faculty of Pharmacy, Tehran University of Medical Sciences, Tehran, Iran. *; b *The Institute of Pharmaceutical Sciences (TIPS), Tehran University of Medical Sciences, Tehran, Iran.*; c *Biomedical Engineering Group, Faculty of Chemical Engineering, Tarbiat Modares University, Tehran, Iran.*; d *Food and Drug Control References Laboratory, Food and Drug Organization, Ministry of Health and Medical Education, Tehran, Iran. *; e *Department of Drug and Food Control, Nanotechnology Research Center, Faculty of Pharmacy, Tehran University of Medical Sciences, Tehran, Iran.*; f *Department of Drug and Food Control and Pharmaceutical Quality Assurance Research Center, Faculty of Pharmacy, Tehran University of Medical Sciences, Tehran, Iran. *; g *Department of Molecular Biology, Food and Drug Control Reference Laboratory, Food and Drug Laboratory Research Center, Food and Drug Organization, Ministry of Health and Medical Education, Tehran, Iran.*

**Keywords:** Recombinant reteplase, Clot lysis, APTT lysis test, Validation, Biological activity

## Abstract

Recombinant plasminogen activator (reteplase) is a third generation thrombolytic agent which has been used on coronary artery thrombosis and acute myocardial infarction. Clot lysis assay is usually considered as a unique method to evaluate biological activity of reteplase. In this study biological activity of reteplase was determined by APTT (activated partial thromboplastin time) lysis method. Validity of this method was evaluated in comparison with reference method, clot lysis time assay. Results of APTT lysis test showed good reproducibility (relative standard deviation (RSD) 3-5% for within day analysis and 4-7% for between day analysis), and accuracy (101.3-102.7%). APTT lysis responses were linear in range of 0.001-0.1 mg/mL reteplase. Therefore, APTT lysis method is applicable for biological activity determination of reteplase. Although more comprehensive studies are required to approve this test as a reference method, APTT lysis method seems to be valuable to receive more attention due to advantages of technical simplicity, sensitivity, applicability, and cost efficiency.

## Introduction

Acute myocardial infarction (AMI) is a common disorder that is often accompanied by significant morbidity and mortality. Thrombolytic therapy, especially by plasminogen activators, is a well-established standard emergency treatment for acute myocardial infarction (AMI) and some other thromboembolic disorders. Among available thrombolytic agents, recombinant plasminogen activator (reteplase, r-PA), has received attention due to its lower side effects, rapid coronary patency, and prolonged half-time compared to other drugs of this group (1). Among various methods for assaying fibrinolytic drugs, those who simulate their physiological function in the body practically represent better *in-vivo, in-vitro* correlation. Thus, clot lysis time methods are usually selected as reference methods in literatures and pharmacopoeias. Several types of lysis time methods have been developed *e.g.* clot lysis time (2, 3), euglobulin clot lysis time (4), ecarin clotting time (ECT) (5), dilute blood clot lysis time (6) and streptokinase activated lysis time (SALT) (7). Clot lysis time methods are based on the reaction of coagulation and fibrinolysis factors present in the medium. Therefore, lysis time represents the time that lysis reactions takes to overcome clotting. According to the monographs of alteplase in the United States Pharmacopeia (USP) as well as British Pharmacopoeia (BP) and European Pharmacopoeia (EP), purified reagents are used in the clot lysis assay (2, 3) and the number of factors and reactions included in the assay are preferably limited to inevitable factors (fibrinogen, thrombin and plasminogen) to reduce costs. The kinetics of the reactions is also simplified by limiting the interfering parameters engaged in the assay. In other methods, however, all or most of the factors participate in coagulation and lysis reactions in body are present. Obviously, it is impossible to use all coagulation and lysis factors in purified form for *in-vitro* analysis thus blood or its derivatives such as plasma and euglobulin fraction of plasma are included in the tests. However, despite their advantage of simulating biological condition, kinetics of the reactions and interpretation of the results are more complicated. Also, there are several uncontrollable parameters that may influence the results. So, to obtain validated results, it is necessary to design these types of methods more deliberately and consider more carefully about influencing factors. 

In the present study, we used plasma as a carrier of coagulation and lysis factors. As suggested in previous study (8), APTT (activated partial thromboplastin time) reagent and CaCl_2_ were used as starters of coagulation reactions in the intrinsic pathway of coagulation cascade (9). APTT test is a familiar method that is used for biological activity of heparin in pharmacopoeias (10). The presence of heparin delays the clotting time of plasma ending with faint lysis due to the fact that heparin only affect coagulation factors (anti thrombin) but lacks fibrinolytic activity (effect on plasminogen). Therefore in determination of heparin, clotting time is monitored while for reteplase (or other plasminogen activators), lysis time of plasma is assessed. The monograph for biological assay of reteplase in the universally accepted pharmacopoeias such as USP, EP, and BP has yet to be established. We therefore used Clot lysis time as a reference assay which has been accepted by pharmacopoeias for alteplase. To our knowledge, APTT lysis method has not been used for determination of biological activity of reteplase (11). We hence determined and compared the potency of reteplase using both clot lysis time and APTT lysis methods. 

## Experimental

*Reteplase*

Commercial reteplase Rapilysin^®^ (Roche diagnostic GmbH, Mannheim, Germany) as standard and Retelies^®^ (Osvah Pharmaceutical Company Tehran, Iran) as sample were purchased as lyophilized powder. Content of each vial was mixed with sterile water to concentration 1.8 mg/mL as manufacturer›s instructions, aliquoted and stored at −20 °C. 

*APTT reagent*

Activated partial thromboplastin time reagent (APTT) and CaCl_2_ were obtained as Hemosil® kit from Instrumentation Laboratory Company (Milan, Italy).

*Blood collection and plasma preparation*

Blood samples were collected by venipuncture without venous occlusion in tube containing 3.8% (w/v) sodium citrate resulting in 1:9 ratio of anticoagulant solution to sample. Plasma was harvested following centrifugation to sediment platelets. Hemolyzed plasma was rejected. The contents of plasma from 6 healthy, normal male/female people were mixed, then aliquoted and stored at -70 °C as pooled plasma. 

Other reagents were purchased from Sigma (St. Louis, MO, USA) or Merck (Darmstadt, Germany). 

In the day of analysis, the aliquots of reteplase and plasma were thawed. The remaining amounts discarded following completion of each experiment.

*Clot lysis time assay*

To a set of glass test tubes, definite volumes of thrombin solution 33 IU/mL (Thrombin from bovine plasma, Sigma, USA) and reteplase standard solution (0.001-0.1 mg/mL) were mixed on ice. In another set of tubes, plasminogen 1 mg/mL (Plasminogen from human plasma, Sigma, USA) and fibrinogen 2 mg/mL (Fibrinogen from human plasma, Sigma, USA) were mixed. Definite volumes from the first series of tubes, transferred to the second series. The time interval between this time and complete lysis time of the clot in the mixture was determined for each concentration of reteplase standard. Calibration curve was plotted by log lysis time *vs.* log reteplase standard concentration (2).

*APTT lysis assay*

In a set of glass test tubes definite amount of plasma and reteplase standard solution (0.001-0.1 mg/mL) were mixed and incubated for 90 sec in circulating water bath 37 °C. APTT reagent was added then incubated for 3 min. Clotting reactions were initiated by addition of CaCl_2 _0.025 M. The tubes were placed in 37 °C circulating water bath. Lysis time for each vial was determined. Logarithm of lysis times as seconds were plotted against logarithm of different concentrations of reteplase standard solutions (0.001, 0.003, 0.01, 0.03, and 0.1 mg/mL). Buffer containing 1.38 g/L of sodium dihydrogen phosphate monohydrate, 7.10 g/L of anhydrous disodium hydrogen phosphate, 0.20 g/L of sodium azide and 0.10 g/L of polysorbate 80 was used for dilution of standard and sample solutions (8).

Microcon centrifugal filter tubes with cut-off 10000 Da were used to filter reteplase of excipients in the sample and standard solutions. Filtered solutions were expected to consist of excipients of these preparations. These filtered solutions were used for evaluation of excipients interactions in APTT lysis method. 

*Statistical analysis*

The graph preparation was performed using Microsoft Excel 2007 and Statistical analysis was carried out by the one-way ANOVA, using the Statistical Package for Social Sciences (SPSS^®^, version 17.0) programs. The data were expressed as mean ± SD (standard deviation). A 0.05 level of probability was taken as the level of significance and 95% level was considered for confidence intervals.

## Results and Discussion

**Figure 1:**
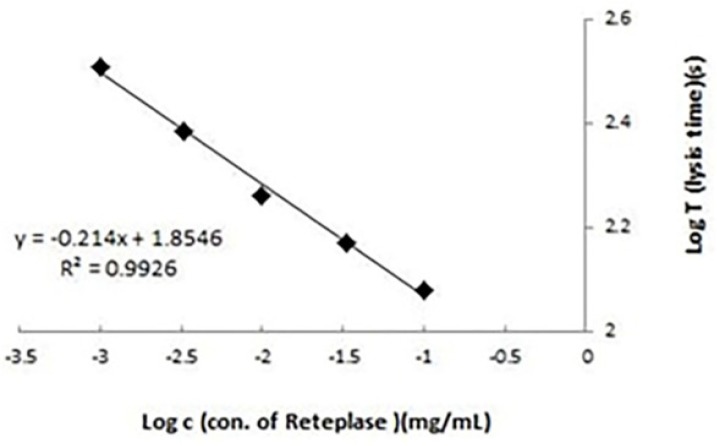
Calibration curve for activated partial thromboplastin time (APTT) lysis test. Lysis times (seconds) were plotted against different concentrations (0.001-0.1 mg/mL) of reteplase standard. Results were the averages of three replicates. Determination of biological activity of recombinant reteplase using clot lysis time and activated partial thromboplastin time (APTT) lysis methods: a comparative study

In the present study, the activity of Retelies^®^ as sample was determined against Rapilysin^®^ as standard by two different methods. Clot lysis time was used as reference method and validity of APTT lysis test was evaluated by comparing the results of this method with the reference method. In APTT lysis test, dilution buffer was used as blank. No lysis reaction was observed using blank sample. Also, the excipients showed to have no interaction with APTT lysis test. Linearity of APTT lysis method was evaluated in the range of 0.001-0.1 mg/mL of reteplase. The standard curve of lysis times against different concentrations of reteplase plotted in a log-log scale showed to be linear (Figure 1). LOD (limit of detection) of APTT lysis method was about 0.0003 mg/mL and quantification limit was 0.001 mg/mL. To determine precision of APTT lysis method, repeatability was assessed using three different concentrations 0.001, 0.01, and 0.1 mg/mL (low, medium and high concentrations respectively) of reteplase and five replicates for each concentration in a day. The RSDs of replicates depicted in Table 1 were about 3.2-5% (within day precision). To evaluate intermediate precision, variation of results in different days (three days, three concentrations of reteplase, and three replicates for each concentration) was calculated as RSD for replicates of each concentration (Table 1, between days precision). Results showed that the method had good repeatability and intermediate precision.

**Table 1:** Precision of activated partial thromboplastin time (APTT) lysis method in two levels (repeatability, within day) and (intermediate precision, between days) for three different concentrations of reteplase (0.001, 0.01, and 0.1 mg/mL)

	**Concentration of reteplase**	**RSD** *
Within day (n:5)	0.1 mg/mL	3.7%
	0.01 mg/mL	3.2%
	0.001 mg/mL	5%
Between days (n:3)	0.1 mg/mL	4.3%
	0.01 mg/mL	4.8%
	0.001 mg/mL	7.2%

* RSDs were calculated based on five replicates for each concentration of reteplase in within day precision and three replicates for between days precision (RSD: relative standard deviation).

In APTT lysis test, it was presumable that plasma as a reservoir of coagulation and lysis factors may influence the results significantly. To eliminate plasma variations, it was reasonable to design the method by pooled normal plasma. To evaluate more comprehensively about the influence of plasma variations and the preference of using pooled normal plasma instead of individual normal plasma, as shown in Table 2, the potency of one definite sample of reteplase was determined in 0.01 mg/mL concentration by different plasma samples from 10 volunteers (All volunteers were healthy female/male people in the range of 20-65 years and with no history of haemostatic disorders). The RSD of tests was 4.1% and found to be in the range of within day repeatability results (3.2-5%) indicating that the effect of plasma variation in discrepancy of the results was negligible. Accuracy of the method depicted in Table 3, was evaluated by comparing the results for three different batches of Retelies^®^ by APTT lysis and reference method (clot lysis time). A regression of results obtained with APTT lysis method versus results obtained with reference method led to a linear graph comparable to the theoretical line with slope 1 and intercept 0 at 95% confidence level. These results indicated that the two methods were comparable to each other and accuracy of APTT lysis method was within the acceptable limit.

**Table 2:** Potency results by different plasma samples (five replicates for each sample)

**Plasma sample**	**APTT lysis assay**
Plasma 1	94.7 ± 3.5%
Plasma 2	97.1 ± 3.1%
Plasma 3	94.0 ± 4.2%
Plasma 4	92.0 ± 4.5%
Plasma 5	98.0 ± 2.9%
Plasma 6	97.8 ± 3.3%
Pooled plasma of plasma 1-6	95.7 ± 3.4%
Plasma 7	107.0 ± 4.0%
Plasma 8	100.2 ± 3.5%
Plasma 9	95.8 ± 4.2%
Plasma 10	94.2 ± 3.6%

**Table 3:** Potency results of three different batches of Retelies^®^ by clot lysis time and APTT lysis method

**Sample**	**Clot lysis time assay**	**APTT lysis assay**
Batch 1	93.1 ± 4.2%	95.7 ± 3.1%
Batch 2	94.7 ± 3.6%	96 ± 2.9%
Batch 3	95 ± 3.2%	97 ± 3.5%

In theory, APTT method is applicable for determination of all anticoagulant and thrombolytic drugs influencing on factors involve in intrinsic, common coagulation pathways or fibrinolytic processes. But studies in this regard showed limitations especially about linearity of APTT results for some of these drugs. For instance, unlike heparin and lepirudin, there is weak linear correlation for warfarin. Favaloro *et al.* and also Lindahl *et al.* have reported that the APTT method could be used for the new generation of antithrombotic agents such as dabigatran, a direct thrombin inhibitor, but the linearity could be lost in higher concentrations of the drug (12, 13). Linear relationship of lysis times and relatively wide ranges of reteplase concentrations (0.001-0.1 mg/mL) in the current study tends to suggest that APTT lysis test is an efficient method for determination of biological activity of reteplase. Although compared to those methods which require either a cocktail of plasminogen, fibrinogen and thrombin (2, 14) or chromogenic substrate (15, 16), the APTT lysis assay showed to be a reliable, short time and cheap method, it is far from a fully developed method for assessing biological activity of reteplase.

## Conclusion

The validity of APTT lysis method was evaluated against clot lysis time as a reference method. Biological activity test using APTT lysis assay was a short-time method and could be suitable for large numbers of samples. Contrary to some other antithrombotic agents, determination of biological activity of reteplase using APTT lysis assay showed to have a good dose-response correlation. APTT lysis assay proved to be a reliable, short-time, and budget-saving method. 
